# HUMAN ALVEOLAR MACROPHAGE FUNCTION IS IMPAIRED IN TUBERCULOSIS CONTACTS WITH DIABETES

**DOI:** 10.21203/rs.3.rs-5489046/v1

**Published:** 2024-11-28

**Authors:** Léanie Kleynhans, Carine Kunsevi-Kilola, Happy Tshivhula, Tariq Webber, Alana Keyser, Nicole Prins, Candice I Snyders, Ayanda Shabangu, Virginie Rozot, Martin Kidd, Hao Zhang, Hong Cai, Yufeng Wang, Adam D Ewing, Stephanus T Malherbe, Abul K Azad, Eusondia Arnett, Blanca I Restrepo, Larry S Schlesinger, Katharina Ronacher

**Affiliations:** 1 DSI-NRF Centre of Excellence for Biomedical Tuberculosis Research, SA MRC Centre for TB Research, Division of Molecular Biology and Human Genetics, Department of Biomedical Sciences, Faculty of Medicine and Health Sciences, Stellenbosch University, Cape Town, South Africa; 2 Mater Research Institute – The University of Queensland, Translational Research Institute, Brisbane, QLD, Australia; 3 Australian Infectious Diseases Research Centre, The University of Queensland, Brisbane, QLD, Australia; 4 Vaccines for Africa, Faculty of Health Sciences, University of Cape Town, Cape Town, South Africa; 5 South African Tuberculosis Vaccine Initiative, Institute of Infectious Disease and Molecular Medicine, and Division of Immunology, Department of Pathology, University of Cape Town, Cape Town, South Africa; 6 Centre for Statistical Consultation, Stellenbosch University, Stellenbosch, South Africa; 7 Department of Molecular Microbiology and Immunology, South Texas Center for Emerging Infectious Diseases, University of Texas at San Antonio, San Antonio, TX, USA; 8 Texas Biomedical Research Institute, San Antonio, TX, USA; 9 Department of Epidemiology, School of Public Health-Brownsville Campus, University of Texas Health Science Center at Houston, Brownsville, TX, USA; 10 South Texas Diabetes and Obesity Institute, School of Medicine, University of Texas Rio Grande Valley, Edinburg, TX, USA

**Keywords:** tuberculosis, type 2 diabetes, mycobacterial growth, alveolar macrophages, monocyte derived macrophages, bronchoalveolar lavage

## Abstract

Type 2 diabetes (T2D) increases susceptibility to tuberculosis (TB) with the underlying mechanisms remaining unknown. To determine whether immune dysfunction in the lung contributes to TB susceptibility, we obtained paired human alveolar macrophages (HAMs) and monocyte-derived macrophages (MDMs) from TB-exposed individuals with/without T2D. Upon infection with *Mycobacterium tuberculosis* (*M.tb),* T2D-HAMs had more *M.tb* growth and produced more TNF. There were fewer neutrophils in the bronchoalveolar lavage of T2D patients which was inversely correlated with *M.tb* growth. Both T2D-HAMs and MDMs expressed less CD32, with T2D patients having fewer M1-like MDMs. T2D-MDMs produced less IL-1RA and CSF2. Overall *M.tb*-induced gene expression was delayed in T2D-HAMs, but genes involved in negative regulation of neutrophil migration were upregulated. T2D-HAM DNA was hypermethylated compared to control HAMs, however genes linked to TNF signalling were hypomethylated. We show here the first in-depth analysis of T2D-HAMs providing an explanation for more severe TB in T2D patients.

## INTRODUCTION

Tuberculosis (TB) remains a leading cause of illness and death globally (1). Patients with type 2 diabetes (T2D) are more likely to develop active TB disease, to present with more severe TB and to experience adverse treatment outcomes (2, 3). Up to 15% of TB cases, globally, are attributed to diabetes (4). Low- and middle-income regions with the fasted growing number of people with diabetes also have the highest TB incidence rates (1, 5). Increased susceptibility to TB in T2D has been attributed to different factors, including the direct effects of hyperglycemia and insulin resistance and indirect effects on macrophage and lymphocyte function (6, 7). However, the underlying mechanisms responsible for the immune dysfunction at the site of infection, the lung, remain unknown.

Upon entering the lung, alveolar macrophages (AMs) are the primary innate immune cells that encounter *Mycobacterium tuberculosis* (*M.tb*) (8). AMs serve as both a host cell reservoir for *M.tb* as well as the effector cell eliminating or controlling *M.tb* (9–13). The efficiency with which AMs kill or inhibit the replication of ingested bacilli is crucial in preventing *M.tb* infection and TB disease. Murine studies demonstrate that during early *M.tb* infection there is a delay in monocyte/macrophage recruitment into the lung of dysglycemic mice due to alterations in the oxysterol/GPR183 pathway (14), which is important in immune cell positioning and granuloma formation in the lung. Alterations in innate responses to *M.tb* during diabetes result in a delay in the delivery of *M.tb* antigens to the lung draining lymph nodes by antigen presenting cells (15). Inefficient phagocytosis, recruitment of macrophages and antigen presentation subsequently delay the activation of the adaptive response, resulting in an increased bacterial load and more severe TB disease in diabetic mice (16). Given the central role of AMs during the early response to *M.tb*, a better understanding of disturbances in AM function in diabetes patients infected with *M.tb* is needed.

Monocytes from diabetes patients (vs. healthy controls) have reduced HLA-DR expression and phagocytosis of *M.tb*, which has been attributed to alterations in the monocyte itself as well as in serum opsonins (17–20). There is however limited information on the effects of T2D on monocyte-derived macrophage (MDM) function. Although some show that high glucose affects MDM function *in vitro* (21), others suggest there are intrinsic functional changes in diabetic macrophages (22).

We currently lack information on the impact of diabetes on human AMs (HAMs) during *M.tb* infection, and whether changes in HAM function correlate with peripheral MDM function in the same individuals. Bronchoalveolar lavage (BAL) samples from diabetes patients are difficult to obtain. Given our team’s expertise in performing bronchoscopies, we collected BAL fluid (BALF) from TB contacts with/without diabetes in South Africa and simultaneously generated MDMs from PBMCs from the same patients. This allowed us to assess alterations in the phenotype and function in HAMs and MDMs, including their ability to phagocytose and limit *M.tb* growth. We show that diabetes has a profound effect on HAM function and that alterations in DNA methylation, early gene expression and cytokine responses to *M.tb* are associated with reduced capacity of T2D-HAMs to contain *M.tb*. These studies advance our understanding of immune dysfunction in the lung during T2D and provide insight into the mechanisms contributing to the increased susceptibility to TB in T2D patients without the confounding impact of active TB disease.

## METHODOLOGY

### Ethics statement

Ethical approval was obtained from the Health Research Ethics Committee of the University of Stellenbosch (N13/05/064A) and was conducted according to the Helsinki Declaration and International Conference of Harmonization guidelines. Written informed consent was obtained from all study participants.

### Study participants

Patients with pulmonary TB (positive Xpert MTB/RIF assay (Cepheid Inc.) and *M.tb* culture) were identified at public healthcare clinics around Cape Town, South Africa (23). Their adult close contacts (>5h/day within a closed space with the symptomatic TB patient) aged 30–65 years were invited to this study. Exclusion criteria included active TB (ruled out based on lack of TB symptoms, normal chest x-ray and negative sputum culture), body-mass index (BMI) <20, positive for HIV, T1D, cancer, pregnancy or breastfeeding, a history of current or recent infections and use of immune modulatory drugs (glucocorticoids, TNF inhibitors, etc.) within the past three months. Documentation of medications taken within the previous month for T2D, or any other health condition was based on self-reporting. Dyslipidemia was assessed by clinical laboratory testing. HbA1c was determined with an automated HemaCue^®^ 501 point-of-care testing system and confirmed by a diagnostic laboratory HbA1c measurement. No-T2D controls (n=11) with HbA1c ≤5.7% and normal blood glucose [random (RBG) <11 mmol/L (<200mg/dL) or fasting blood glucose <6.1mmol/L (<110mg/dL)] and patients with T2D (n=12) with HbA1c ≥7%, and/or newly diagnosed or known T2D and/or high fasting plasma glucose (≥7mmol/L / 126mg/dl) were included in the study. Endogenous insulin and cortisol were measured by the National Health Laboratory Service. QuantiFERON-Gold In-Tube assays were performed (Qiagen) as an indication of latent TB status.

### Bronchoalveolar lavage

**Figure 1.**
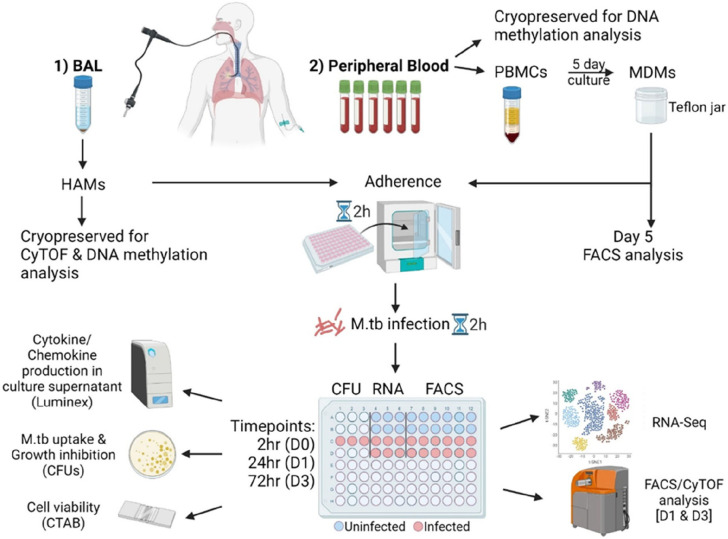
Graphical summary of the methodology employed to process samples collected from all study participants. 1) BAL cells were cryopreserved for CyTOF and DNA methylation analysis, while some were infected with *M.tb* on the day of collection. 2) In parallel, blood collected in NaHep tubes was used to isolate PBMCs. PBMCs were cultured in autologous plasma for 5 days in Teflon jars to allow monocytes to differentiate to MDMs. On day 5, MDMs were collected for flow cytometric analysis and for the MDM stimulation assay. HAMs and MDMs were allowed to adhere to the tissue culture plates for 2h. They were subsequently infected for 2h with *M.tb* H_37_R_v_ (MOI 1:1) for 2h and cultured for 1 and 3 days. Culture supernatants were collected for the Luminex analysis and cells collected for CFU determination, RNA sequencing and FACS (MDMs) or CyTOF (HAMs) analysis. Cell viability was determined using the CTAB method. This figure was created with BioRender.com.

BAL was performed using a fiberoptic bronchoscopy. Two hundred ml of saline solution was instilled in aliquots of 50ml each and aspirated to collect BALF from the lower airways and alveoli. Upon collection, 2ml of BALF was sent to the National Health Laboratory Service to perform cytospins and May-Grunwald Giemsa staining to determine differential cell counts. Approximately 90% [95% CI=80–95 (no-T2D) and 70–95 (T2D)] of cells were macrophages (supplementary figure S1a). Cytology was performed by microscopy using a Zeiss Axioskop 2 Plus Ergonomic Trinocular Microscope with Zeiss ZEN lite v2.0 software. The remaining BALF was centrifuged at 200×g for 10min at 4°C to pellet and wash cells with RPMI-HEPES (Sigma-Aldrich). Cells were resuspended in RPMI (Biowest) containing 2mM glutamine (Sigma-Aldrich), counted and plated for the stimulation assay (Figure 1).

### Peripheral blood mononuclear cell isolation and MDM differentiation

From the same participants peripheral blood mononuclear cells (PBMCs) were isolated from heparinized whole blood as described in the supplement.

### Mycobacterial growth inhibition assay

MDMs and HAMs were cultured in 96-well flat bottom tissue culture plates at a density of 1.3×10^5^cells/well in RPMI containing 2mM glutamine and 20% human AB serum (Celtic Molecular Diagnostics) (Figure 1). After a 2h adherence step, non-adherent cells were eliminated by washing with warm RPMI-HEPES. MDMs and HAMs were infected with the H_37_R_v_ MA strain (ATCC: 27294) for 2h (MOI=1). The cell viability and colony forming units (CFUs) were assessed at 2h, day 1 (D1) and day 3 (D3) post infection (p.i.) as described in the supplement.

### Cytokine quantification using Luminex

Culture supernatants of uninfected and infected MDMs and HAMs were collected at all time points. Supernatant collected at 2h was replaced with fresh medium, followed by incubation until D3. Supernatants were filter sterilized using 4mm Millex^®^ filter units (0.22μm) with low protein binding PVDF membranes (Merck) and stored at −80°C. The concentrations of 13 analytes were measured using a Merck Human Cytokine/Chemokine Magnetic Bead Panel (Merck-Millipore) as described in the supplement.

### Flow cytometry and CyTOF analysis

MDMs taken from the Teflon jars (day 5) as well as uninfected and infected MDMs retrieved from the 96-well plates, using 5mM EDTA, at D1 and D3 p.i. were stained using two flow cytometry panels as described in the supplementary materials (supplementary table S1). A time-of-flight mass cytometry (CyTOF) panel was designed to characterize the HAM phenotypes (supplementary table S2). Samples from each participant were barcoded, pooled and stained as described in the supplement.

### RNA isolation and sequencing

Using 5mM EDTA, uninfected and infected MDMs and HAMs were retrieved from the 96-well plates at each time point. Cells were stored in RNALater (Invitrogen) at -80°C. Prior to shipment all samples were transferred to TRIzol (ThermoFisher). RNA was isolated using the Quick-RNA Microprep Kit (Zymo Research) according to the manufacturer’s instructions. The RNA quality and quantity were assessed, the libraries prepared, and the sequencing done as described in the supplement.

### RNA sequencing data preprocessing and analysis

The RNA-Seq data analysis was done using CLC Genomics Workbench 22 (Qiagen) as described in the supplement.

### DNA extraction and methylation profiling

Genomic DNA was isolated from unstimulated whole blood and BAL cells, as described in the supplement.

### Methylation data preprocessing and analysis

Analysis of methylation arrays was carried out following the Bioconductor ‘methylationArrayAnalysis’ workflow (24) as described in the supplement.

### Statistical analysis

Data were analyzed using GraphPad Prism v9. For two group comparisons, the Mann-Whitney U test was used for non-parametric data and the Studenťs t test for parametric data. For paired analyses the Wilcoxon matched pairs signed rank test was used. Categorical data were analyzed using the Fisher Exact test. Correlations were done with a Spearman rank test. Luminex data were transformed to correct for the distribution of the data. Analytes were Winzorised or BoxCox transformed using Statistica v14 (StatSoft). The Holm-Šídák method was used to correct for multiple comparisons when using a one-way ANOVA and the Šídák’s multiple comparison test when using a two-way ANOVA. For both approaches an adjusted p<0.05 was considered significantly different. Heatmaps were generated using the ComplexHeatmap package (25) in R (26). IPA was used to generate canonical pathways as well as diseases functions associated with different cytokines clades from the heatmap. The circlize package (27) in R was used to generate a chord diagram using pathways with the lowest p-values.

## RESULTS

### Participant characteristics

Ninety-five contacts were enrolled into the ALERT parent study after screening 247 participants for T2D (23). Of these contacts, 11 without T2D (no-T2D) and 12 with T2D (T2D), who met the inclusion criteria, agreed to undergo a bronchoscopy and venipuncture. All participants were of the mixed ancestry South African Colored ethnicity. The two groups did not differ in age or smoking status and were predominantly females (table 1). Contacts without T2D were all QFT positive, while half of the T2D group was QFT negative. Of the factors frequently associated with T2D, only BMI and triglycerides were significantly higher in T2D patients. Ten of the T2D patients had previously been diagnosed with diabetes and were using statins or sulfonylureas or a combination of drugs (statins, sulfonylureas, insulin and/or hypertensive drugs). One no-T2D control used medication for hypertension.

### Human alveolar macrophages from T2D patients have a reduced ability to control *M.tb* growth

**Figure 2.**
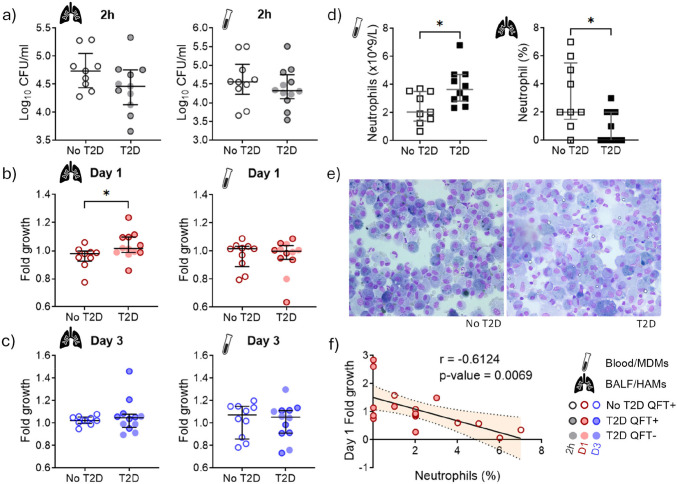
Early interactions between *M.tb* and human alveolar macrophages are impacted by T2D status. Human alveolar macrophages (HAMs) and monocyte-derived macrophages (MDMs) from contacts of TB patients with and without T2D were infected in parallel with *M.tb* H_37_R_v_ (MOI 1:1) for 2h and subsequently cultured for 1 and 3 days. a) Number of bacilli, presented as log_10_ CFU/ml, associated with HAMs and MDMs after 2h. Bacterial load, presented as fold growth, in HAMS and MDMs cultured for b) 1 and c) 3 days. d) Neutrophil numbers measured in whole blood (absolute count) and BALF (percentage). e) Pictures of representative cytospins from BALF of contacts without T2D (left) and with T2D (right) taken at a 40x magnification. f) Correlation between neutrophil percentage in BALF and day 1 *M.tb* fold growth in HAMs. Data are presented as medians with interquartile ranges. CFU – colony forming units. *: p < 0.05

To determine whether diabetes impacts *M.tb* phagocytosis and growth in HAMs and MDMs, we infected the cells with *M.tb* for 2h, and continued to culture the cells for up to 3 days. There was no significant difference in the number of *M.tb* bound/phagocytosed by HAMs and MDMs of T2D patients (versus no-T2D) after 2h (figure 2a) nor were there differences in the CFU counts at D1 and D3 p.i. (supplementary figure S3a,b). After correcting for the number of *M.tb* bound/phagocytosed by the macrophages at 2h, no differences were seen in the adaptation of *M.tb* to the intracellular environment (D1 p.i.) and bacterial growth (D3 p.i.) in MDMs of T2D patients when compared to no-T2D (figure 2b,c). HAMs from T2D patients, however, had a significantly higher *M.tb* fold growth at D1 p.i. (figure 2b), irrespective of QFT status (supplementary figure S3c,d). Cell number did not affect the CFU data as the number of viable cells were equal across conditions for both MDMs and HAMs (supplementary figure S3e). The number of viable cells remained constant over the 3-day period for HAMs, while there was an overall reduction of 10% in MDMs for both patient groups.

Of the parameters evaluated in the blood, hyperglycemia (HbA1c; spearman r0.4380, p=0.0470) was associated with *M.tb* fold growth in HAMs at D1 p.i. (table 2). There was a trend for MDM CFU counts to be associated with LDL (2h p.i.; Spearman r0.3632, p=0.0885) and HDL concentrations (D3 p.i.; Spearman r0.4053, p=0.0550) (supplementary table S4). Differentiation of the MDMs was carried out in medium containing 20% autologous serum and therefore the results suggest that lipids may impact bacterial control.

### Neutrophil counts in BALF are lower in T2D and inversely correlated with *M.tb* growth in HAMs

Complete blood counts and differential cell counts were done to assess changes in cell numbers by T2D status. The absolute white cell and neutrophil counts were significantly higher in T2D when compared to no-T2D (figure 2d and supplementary figure 1b). In contrast to blood, the percentage of neutrophils in the BALF was significantly lower in T2D (figure 2d,e). No differences in the percentage of macrophages and lymphocytes were observed in the BALF between the two groups (supplementary figure 1a). The neutrophil percentage in BALF was negatively correlated (Spearman r-0.6124, p=0.0069) with the *M.tb* fold growth in HAMs at D1 (figure 2f). This suggests that T2D-induced changes in HAM responses may impair neutrophil recruitment to the site of infection and highlights the importance of a macrophage-neutrophil interaction in the lung.

### Reduced frequency of M1-like MDMs and downregulation of CD32 in HAMs and MDMs from T2D patients

**Figure 3.**
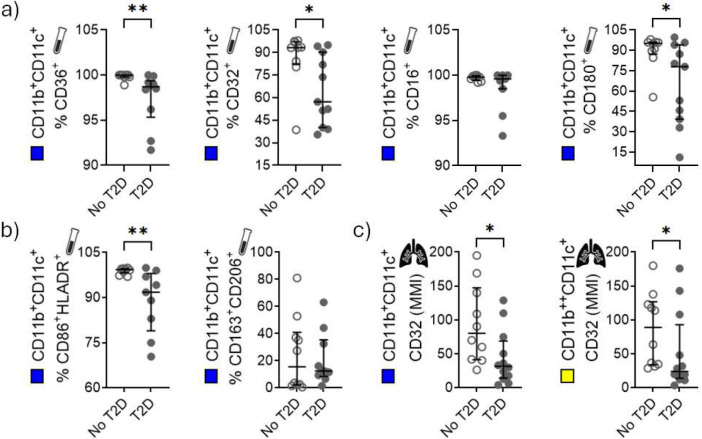
Frequencies of MDMs expressing CD36, CD32, CD16 and CD180 by T2D status. PBMCs were cultured in Teflon jars for 5 days to allow monocytes to differentiate into MDMs. After 5 days the PBMCs were stained for flow cytometric analysis. In addition, cryopreserved cells obtained from the BALF of the same participants were stained for CyTOF analysis. a) Frequencies of CD11b^+^CD11c^+^ (blue) MDMs expressing different cell surface markers. b) MDMs expressing cell surface markers associated with M1-like (CD86, HLA-DR) and M2-like (CD163, CD206) cells. c) CD32 expression in CD11b^+^CD11c^+^ (blue) and CD11b^++^CD11c^+^ (yellow) HAM populations. Data are presented as medians with interquartile ranges. *: p<0.05, **: p<0.01

We continued to investigate whether the expression of the scavenger receptor CD36, Fcγ receptors CD16, CD32 and CD64, the chemokine receptor CCR2 and CD180 vary by T2D status in MDMs and HAMs. Differentiated MDMs were subjected to flow cytometric analysis, prior to infection, and consisted of approximately 50% of CD11b^+^CD11c^+^ cells, 20% of CD11b^−^CD11c^+^ cells and <2% of CD11b^+^CD11c^−^ cells (supplementary figure S4a,b). The composition of macrophage populations was similar in the two patient groups, suggesting that monocyte differentiation or heterogeneity of cells in the population was not altered by T2D status. Given that MDMs express both CD11b and CD11c (28), and that majority of cells were CD11b^+^CD11c^+^, we continued to gate on the individual markers within this population using the unstained and isotype controls (supplementary figure S4c). The frequency of MDMs expressing CD36, CD32 and CD180 was significantly reduced in T2D compared to no-T2D (figure 3a and supplementary figure S5). There was also a reduction in the frequency of MDMs with a M1-like phenotype (CD11b^+^CD11c^+^CD86^+^HLA-DR^+^) in T2D compared to no-T2D (figure 3b), but no difference in MDMs with a M2-like phenotype (CD11b^+^CD11c^+^CD163^+^CD206^+^).

Due to the high autofluorescence of alveolar myeloid cell populations, mass cytometry was used to assess HAM phenotypes. Within the CD45^+^ population in the BAL samples, approximately 70–80% of cells were CD11b^+^CD11c^+^, while approximately 15–20% were CD11b^++^CD11c^+^ (supplementary figure S6a,b). Since majority of myeloid cells in BALF are HAMs and express both CD11b and CD11c (29, 30), we continued to evaluate the expression of the different cell markers in the CD11b^+^CD11c^+^ HAM population. The CD11b^++^CD11c^+^ population was considered to be monocyte-derived/infiltrating cells (31). Both HAMs and infiltrating cells of T2D patients express less CD32 (based on mean metal intensity) when compared to cells of no-T2D controls (figure 3c). No changes were observed in any other marker (data not shown).

MDMs from T2D patients retrieved from the *M.tb* stimulation assay 1 and 3 days p.i. also had reduced frequencies of CD36 expressing cells compared to cells from no-T2D controls, reaching statistical significance at D3 (supplementary figure S7). In *M.tb*-infected cells, there was no difference in frequency in any of the cells (supplementary figure S7). In addition, there were no differences in the expression of any of the cell surface markers, measured by CyTOF, in HAMs subjected to the *M.tb* stimulation assay (data not shown). Taken together, T2D patients have reduced frequencies of M1-like blood derived macrophages and reduced expression of FcγRII (CD32).

### *M.tb*-induced gene expression is delayed in HAMs from T2D patients

**Figure 4.**
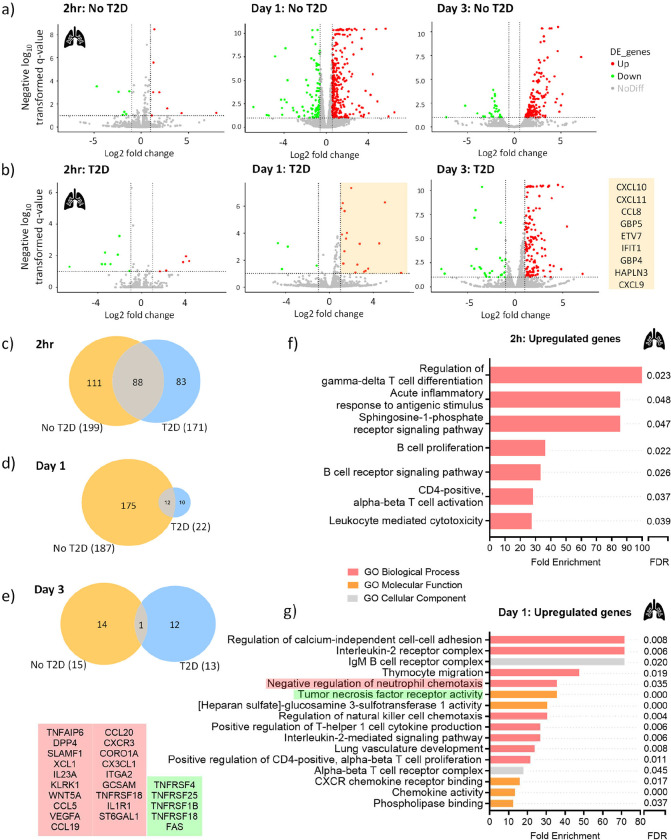
*M.tb*-induced alterations in gene expression are delayed in HAMs of T2D patients. Differential expression analysis of *M.tb*-infected HAMs of a) no-T2D controls and b) T2D patients compared to uninfected HAMs. Genes with FDR-adjusted p-value<0.1 and log2 fold changes (LFC) of >1 or <−1 were considered differentially expressed. Green corresponds to genes whose expression was significantly downregulated upon *M.tb* infection and red corresponds to genes whose expression was significantly upregulated. Venn diagrams were used to illustrate the number of co-expressed genes. Venn diagrams were also used to indicate the number of co-expressed genes between the two patient groups at c) 2h, d) day 1 and e) day 3. Gene Ontology Resource (PANTHER) was used to determine the biological processes, molecular functions and cellular components the genes, that were significantly upregulated in *M.tb*-infected T2D HAMs vs no-T2D HAMs, were involved in at f) 2h and g) day 1.

Next, we sought to analyze the *Mtb*-induced gene expression profiles in HAMs and MDMs. Two hours p.i, only a few genes were up- (8) or downregulated (7) (LFC>1, FDR<0.1) in no-T2D-HAMs in response to *M.tb* (figure 4a, supplementary table S5). The number of DEGs increased at D1 (144 up- and 43 downregulated; supplementary table S6) and remained high at D3 (170 up- and 29 downregulated; supplementary table S7). Sixty-five genes were co-expressed at D1 and D3 (supplementary figure S8a,b) and are involved in the following pathways: IDO1, type 1 IFNs (IFN-α/β), and IFN-γ. In HAMs of T2D patients, little change in gene expression was also observed at 2h (5 up- and 8 downregulated) (figure 4b, supplementary table S8). The gene expression in T2D-HAMs however was delayed at D1 with only 22 DEGs detected (vs 187 in no-T2D-HAMs) (supplementary table S9). An increase in DEGs was observed at D3 (144 up- and 27 downregulated; supplementary table S10) but remained lower than those reported in no-T2D-HAMs (171 vs 199 DEGs). As a result of this delay, only 10 genes were co-expressed at D1 and D3 of T2D-HAMs (Supplementary figure S8c,d). The little overlap in DEGs between the two patient groups at each time point indicates that HAMs from T2D patients have a delayed but also a unique transcriptional profile in response to *M.tb*. (figure 4c,d,e; supplementary figure 9a,b).

Interestingly, canonical pathways that were inhibited in T2D-HAMs (vs no-T2D-HAMs) at 2h, include ‘G-protein coupled receptor signaling’, ‘phagosome formation’, ‘focal adhesion kinase (FAK) signaling’ and ‘CREB signaling’. Of relevance is the involved of these pathways in early cell migration, macrophage activation and function. At the same time point, diseases and biological functions that were inhibited in T2D-HAMs included the ‘development of antigen presenting cells’ and ‘adhesion of granulocytes functions’ which is consistent with the reduced neutrophil count observed in the BALF. At D1 in T2D-HAMs, ‘binding of leukocytes’ was inhibited whereas in no-T2D-HAMs ‘apoptosis of phagocytes’, ‘apoptosis of myeloid cells’, ‘apoptosis of macrophages’, ‘apoptosis of MDMs’ and ‘differentiation of macrophages’ were activated. In contrast to the apoptotic pathways that were activated in no-T2D-HAMs, ‘necrosis’ and ‘cell death of immune cells’ were functions that were activated in T2D-HAMs at D3. Suggesting a shift towards unprogrammed cell death in HAMs of T2D patients.

The GO biological processes, molecular functions and cellular components in which the genes that were significantly upregulated in *M.tb*-infected T2D-HAMs (vs no-T2D-HAMs) were involved in at 2h included processes involved in the activation of the adaptive immune response (figure 4f). More importantly, the genes that were upregulated in T2D-HAMs at D1 were involved in ‘tumor necrosis factor receptor activity’ and ‘negative regulation of neutrophil chemotaxis’ (figure 4b,g). These results are consistent with the decreased number of neutrophils observed in BALF from T2D patients (figure 2d,e) and the increased TNF produced by HAMs at this time point (figure 7c).

Due to low participant numbers and low RNA yield, the RNA-Seq analysis of MDMs of no-T2D controls could not be performed. The gene expression pattern of MDMs from T2D patients, were similar to HAMs from no-T2D controls (supplementary figure S10) with little to no co-expressed genes between the HAMs and MDMs of T2D patients (data not shown).

### The kinetics and magnitude of cytokine responses in HAMs and MDMs differ by T2D status

**Figure 5.**
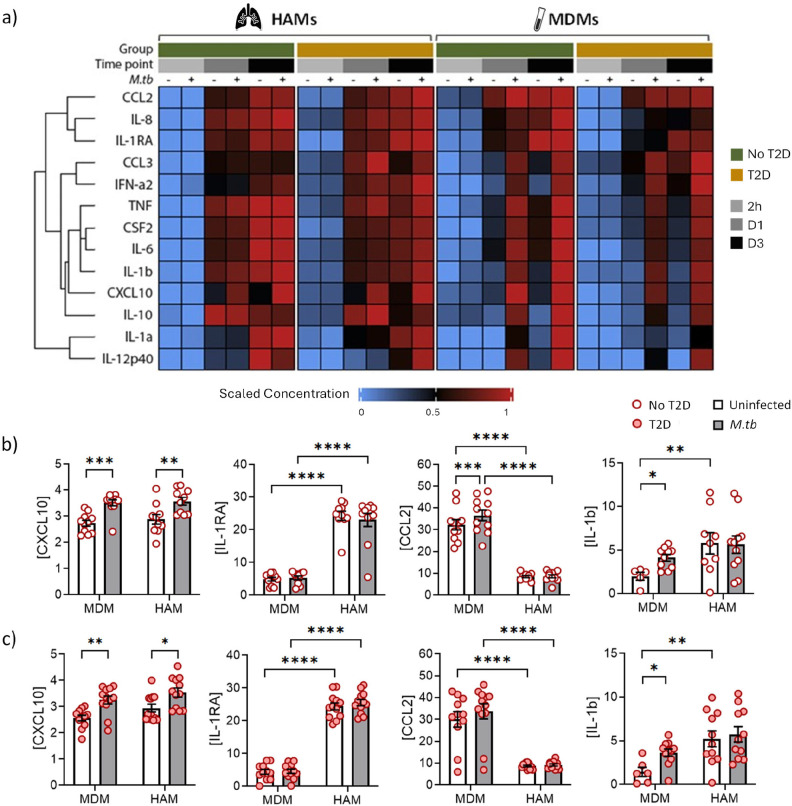
Cytokine/chemokine concentrations in HAMs and MDMs culture supernatant. Culture supernatants from both infected and uninfected cells were collected at 2h, day 1 and 3 p.i. and the cytokine/chemokine concentrations measured using the Luminex platform. a) Protein concentrations were clustered according to cell type, group and time point. The ComplexHeatmap package in R was used to generate the heatmap using the mean concentration of each analyte at each time point. Within each cell type, the analyte means were scaled between 0 (blue) and 1 (red). b) Cytokine concentrations secreted by MDMs and HAMs of no-T2D controls at day 1 p.i. c) Cytokine concentrations from MDMs and HAMs of T2D patients at day 1 p.i. Luminex data were transformed to correct for the distribution of the data. Data are presented as mean±SEM. An adjusted p-value <0.05 was considered significant after correcting for multiple comparisons. CC: close contact, *: p<0.05, **: p<0.01, ***: p<0.001

Before comparing the functional differences of HAMs and MDMs between the two patient groups, we first compared the *M.tb*-induced cytokine/chemokine responses of HAMs and MDMs. Like our previous observation in HAMs, cytokine/chemokine concentrations varied over time (32). Consistent with the findings reported by Tomlinson *et al.* (33), greater differences in the cytokine concentrations between uninfected and *M.tb*-infected culture supernatants were observed in MDMs (D1 and D3 p.i.), than in HAMs (figure 5a). *M.tb* infection significantly increased CXCL10 concentrations in both HAMs and MDMs culture supernatant (in both patient groups), but not IL-1RA concentrations (D1; figure 5b,c). MDMs produced less IL-1RA and more CCL2 and IL-8, compared to HAMs (Figure 5b,c, supplementary figure S11). Similar to the observation in IL-1β concentrations, HAMs produced high basal levels of cytokines.

**Figure 6.**
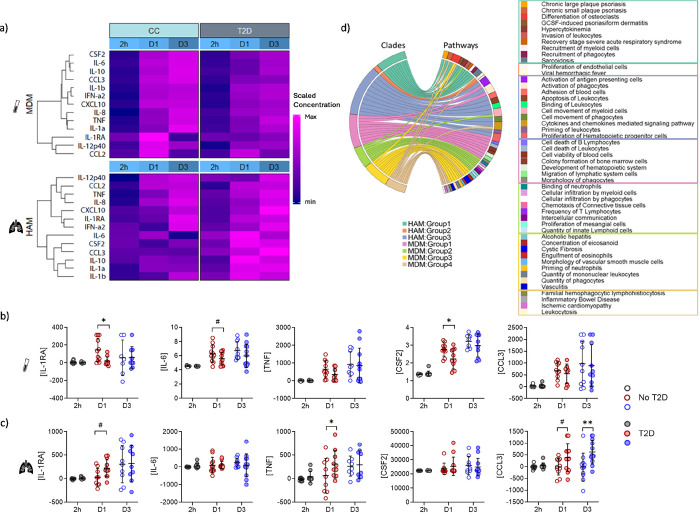
MDMs and HAMs have opposing *M.tb*-induced cytokine responses associated with T2D status. Culture supernatants from both infected and uninfected cells were collected at 2h, day 1 and 3 p.i. and the cytokine concentrations measured using the Luminex platform. *M.tb*-induced cytokine/chemokine concentrations were normalized to uninfected controls. a) Using a heatmap, analyte concentrations were clustered according to cell type, group and time point. Within each cell type, the analyte concentrations were scaled around the median concentration. b) *M.tb* specific cytokine concentrations measured in the culture supernatant of MDMs. c) *M.tb* specific cytokine concentrations measured in the culture supernatant of HAMs. Luminex data were transformed to correct for the distribution of the data and the data presented as medians with interquartile ranges. An adjusted p-value < 0.05 was considered significant after correcting for multiple comparisons. d) Chord diagram summarizing the pathways in which the analytes of the individual clades are involved in.

To assess *M.tb*-induced cytokine responses between no-T2D and T2D we corrected for the baseline response by subtracting the cytokine concentration of uninfected cells from the *M.tb* infected cells. Hierarchical clustering of the data highlighted the diversity in cytokine responses of no-T2D vs T2D cells (figure 6a). *Mtb*-induced cytokine concentrations were increased in MDMs of no-T2D controls at D1 which further increased by D3, except for IL-1RA, IL-12p40 and CCL2. In comparison, cytokine responses in MDMs of T2D patients were lower. The opposite was observed in HAMs. Cytokine concentrations in HAMs of T2D patients were higher at D1 than in HAMs of no-T2D controls. In addition, different groups/clades of cytokines were increased in T2D-HAMs when compared to no-T2D-HAMs (figure 6a). More specifically, concentrations of IL-1RA and CSF2 were significantly lower at D1 for MDMs of T2D patients with a trend for lower IL-6 concentrations (figure 6b, supplementary figure S12a). TNF concentrations were significantly higher in D1 supernatant and CCL3 concentrations significantly higher in the D3 supernatant of HAMs of T2D patients (figure 6c, supplementary figure S12b). There was also a trend for higher IL-1RA and CCL3 concentrations on D1.

Based on the responses over time, cytokines clustered into different clades within each cell population. Pathway analysis on the individual clades of HAMs revealed that the cytokine responses of these cells are involved in endothelial cell proliferation; the movement, adhesion and recruitment of leukocytes and more specifically myeloid cells; the activation of phagocytes and antigen presenting cells and leukocyte apoptosis (figure 6d). MDM responses are also involved in the infiltration of myeloid cells, but more specifically are involved in leukocytosis and the quantity of mononuclear leukocytes, T lymphocytes and innate lymphoid cells as well as the binding and priming of neutrophils. These functions support our findings of increased neutrophil numbers in the blood of T2D patients and decreased numbers in the lung and suggest that neutrophil migration from the blood to the lung is reduced by T2D. MDM responses are furthermore involved in vasculitis which could contribute to the macro or micro-vascular complications associated with T2D.

### T2D affects DNA methylation in HAMs

**Figure 7.**
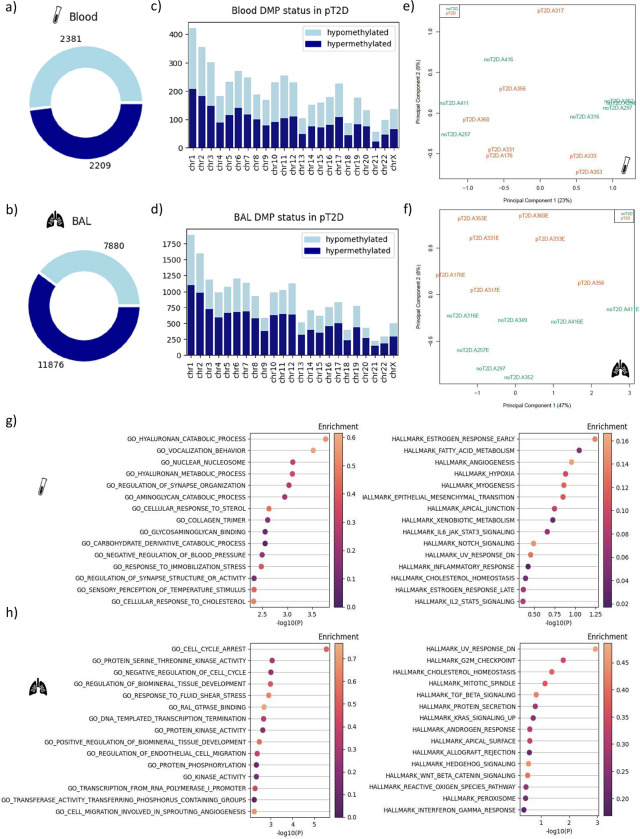
Summary and genomic locations of differentially methylated positions (DMPs) of CpGs (uncorrected p < 0.01). Hyper- and hypomethylation refer to T2D methylation levels relative to non-diabetic levels. Overall hypomethylated and hypermethylated DMP counts are shown for whole blood leucocytes (51.9% hypomethylated) (a) and BAL (39.9% hypomethylated) cells (b). Individual chromosome methylation statistics are shown for whole blood leucocytes (c) and BAL cells (d). Principal component analysis of the methylation profile of individual participants shows no separation by T2D status in the blood (e), and a perfect separation by T2D status in BAL cells (f). Gene set enrichment analysis was done on the whole blood leucocytes (g) and BAL cells (h) using Human MSigDB Collections’ hallmark and gene ontology gene sets. The enrichment score (color gradient on the plots) refers to the fraction of genes in the gene set that were associated with differential methylation in each sample type.

Finally, we assessed alterations in DNA methylation in unstimulated blood leucocytes and BAL cells. Among T2D patients, hypermethylated DMPs were higher in BAL cells compared to blood leucocytes (Fisher’s exact p<2.2×10^−16^; figure 7a,b) and the increase in methylation was evident across all chromosomes (figure 7c,d). The fraction of DMPs across different genomic locations and gene components were however the same between the two sample types (supplementary figure S2b,c). The DNA methylation profiles in the BAL cells separated patients by T2D status, but not in the blood leucocytes (figure 7e,f). Based on the gene set enrichment of the blood leucocyte data, genes involved in cellular responses to sterols, and early estrogen response and fatty acid metabolism are differentially methylated (figure 7g). In BAL cells, genes involved in protein kinase activity, regulation of endothelial cell migration and protein phosphorylation as well as cholesterol homeostasis are differentially methylated (figure 7h). Given the variation in the number of hypo- and hypermethylated genes in the BAL cells, we assessed the gene sets that were either hypo- or hypermethylated in this sample type. Genes involved in the TNF response (PHONG_TNF_RESPONSE_NOT_VIA_P38; FDR=0.0294) as well as the negative regulation of cell proliferation (FDR=0.0418), positive regulation of gene expression (FDR=0.0418) and nucleic acid binding transcription factor activity (FDR=0.0418) had reduced methylation. A decrease in methylation in genes involved in the TNF response in BAL cells is consistent with the activation of the TNF signaling in the transcriptomic analysis and the increase in TNF production in HAMs.

## DISCUSSION

**Figure 8.**
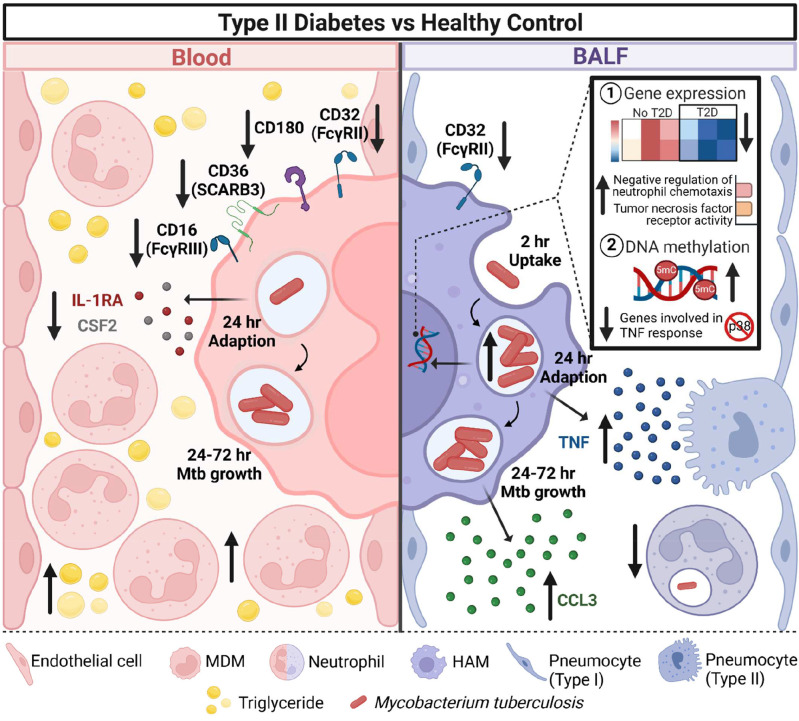
Schematic summary of differences in the response of human monocyte-derived macrophage (MDM) and alveolar macrophage (HAM) to Mtb, by type 2 diabetes status. The blood of T2D patients has higher levels of glucose and triglycerides and circulating white blood cells and neutrophil counts. In contrast, T2D patients have lower neutrophil percentages in their bronchoalveolar lavage fluid. MDMs from T2D patients express less CD32, CD180, CD36 and CD16 and produce less IL-1RA and CSF2 (GM-CSF) in response to *M.tb* infection (day 1 p.i.). However, these changes did not result in changes in the ability of MDMs to phagocytose or kill *M.tb*. HAMs from T2D patients also express less CD32. HAMs, however, produce more TNF (day 1 p.i.) and CCL3 (MIP-1α) (day 3 p.i.) in response to *M.tb*. Twenty-four h (day 1) p.i., HAMs of T2D patients have a significantly higher bacterial burden which coincides with an overall decrease in gene expression and increase in DNA methylation. This figure was created with BioRender.com.

In this study we report that HAMs from T2D patients have a reduced capacity to control *M.tb* growth and that lower BALF neutrophil percentages in T2D are inversely correlated with HAM CFUs (figure 8). T2D patients have lower frequencies of M1-like MDMs while M2-like MDM frequencies remained unchanged. *M.tb*-induced gene expression in T2D-HAMs was delayed. Despite this delay, genes that were significantly upregulated in *M.tb*-infected T2D-HAMs (vs no-T2D-HAMs) are involved in the negative regulation of neutrophil chemotaxis and TNF signaling. Genes involved in the TNF response were furthermore hypomethylated, which all contribute to the increased TNF production in T2D-HAMs. Finally, expression of the FcγRII CD32 was reduced in MDMs and HAMs of T2D patients.

In our investigation, T2D was not associated with reduced *M.tb* binding/phagocytosis in MDMs and HAMs, or reduced containment of *M.tb* in MDMs. Lopez-Lopez *et al.* showed that MDMs of TB-naïve diabetes patients had impaired capacity to associate with *M.tb*, and despite the reduced association, had significantly increased intracellular bacterial loads when infected with a hypervirulent clinical isolate of *M.tb* (vs MDMs of healthy controls) (34). Discrepancies in MDM function, reported here and by others, are likely due to the experimental approach including the isolation of monocytes using magnetic beads and differentiation in CSF2 containing medium for 6/7 days (34, 35) compared to culturing PBMCs in autologous plasma for 7 days, *Mtb* strain and MOI used. Interestingly, we observed that HAMs from T2D patients had an impaired ability to control *M.tb* growth early after infection (D1 p.i.). Wang *et al.* reported that TB patients with T2D have lower proportions of hypodense HAMs, meaning they have a lower magnitude of activation in response to *M.tb* (36). This finding is supported by the HAM RNA-Seq and CFU data presented here where there was a delay in *M.tb*-induced gene expression and a reduction in *M.tb* killing in T2D-HAMs at the earlier timepoint of the stimulation assay. This variation in T2D associated macrophage (MDM vs HAM) responses was also observed in murine macrophage populations, where AM but not bone marrow-derived or resident peritoneal macrophages responses were defective in diabetic animals (15, 37, 38). Therefore, intrinsic effects in AMs contribute to increased susceptibility to TB and to a higher bacterial load in the lungs of mice with diabetes. We now also show that HAMs and not MDMs from T2D patients are defective and that early *M.tb* and host interactions, in the lung, are altered in T2D patients.

We found reduced neutrophil percentages in the BALF of T2D patients and like Raposa-Garcia *et al.* and Andrade *et al.*, increased peripheral white blood cell and neutrophil counts in T2D patients (39, 40). Interestingly we found that genes that were significantly upregulated in T2D-HAMs when compared to no-T2D-HAMs were involved in the negative regulation of neutrophil chemotaxis. Upon infection, macrophages produce mediators (CXCL1 and CXCL2) to recruit neutrophils to the site of infection and crosstalk between these two cell populations is key for the clearance of *M.tb* (41). *M.tb*-infected macrophages activate neutrophils and enhance their effector function (42). Conversely, macrophages acquire antimicrobial peptides through efferocytosis of apoptotic neutrophils or neutrophil granules, resulting in enhanced clearance of *M.tb* in the macrophage (43). These findings suggest that T2D-induced changes in macrophage function impair neutrophil recruitment to the lung, highlighting the importance of the communication between macrophages and neutrophils in immune dysfunction in the lungs of T2D patients.

Like Valtierra-Alvarado *et al*. we found that differentiation of monocytes was not affected by T2D status and that, once differentiated, the MDMs of T2D patients expressed less CD86 and HLA-DR (44). The reduced frequency of CD86^+^HLA-DR^+^ MDMs suggests an inflammatory bias of cells migrating from the periphery to the lung of T2D patients. In keeping with our previous finding, the frequency of MDMs expressing the FcγRII family (CD32) was reduced in T2D patients (18). Also, the amount of CD32 expressed on HAMs from T2D patients was reduced. Since our analysis does not distinguish between the different family members, including FcγRIIA, FcγRIIB and FcγRIIC, and acknowledging the opposing cellular functions of each of these proteins, it is difficult to conclude what implication these changes may have on responses to *M.tb*. FcγRIIA is highly expressed in monocyte and macrophages, while FcγRIIB has low to moderate expression in monocytes and macrophages, respectively (45). While IFN-γ increases the expression of FcγRIIA on monocyte-derived dendritic cells leading to dendritic cell maturation, TNF reduces its expression (46). The increased concentrations of TNF produced by HAMs from T2D patients may therefore result in the reduced expression of FcγRIIA affecting innate immune cell maturation and activation and potentially in a decrease in immune complex clearance in *M.tb* infected hosts.

We furthermore show that HAMs from T2D patients produce more TNF and CCL3, 1 and 3 days p.i., respectively and that the reduction in methylation in genes in the TNF response may contribute to the increased TNF production observed in HAMs infected with *M.tb*. Although the release of TNF in response to mycobacterial infection is important for granuloma formation and for controlling bacterial growth (47), excessive production of TNF and increased sensitivity to the cytokine in tissue have been implicated in TB immunopathology. Excess TNF (relative to its receptor) in BALF of TB patients was associated with tissue necrosis and cavity formation (although no causal relation was proven) (48) and increased immunopathology in TNF knockout mice (49). The trend for an increase in IL-1RA production is likely due the increase in TNF signaling in these cells as TNF induces IL-1RA production (50). Increased TNF concentrations alongside IFN-γ and IL-1β concentrations were measured in lung homogenates from mice with chronic streptozotocin-induced diabetes (vs. euglycemic mice) which coincided with greater areas of inflammation in the lung tissue (15). One possible explanation for impaired control of *M.tb* growth in the HAMs, despite the high level of TNF produced, could be a reduced capacity of lung macrophages to respond to TNF stimulation in T2D patients. MDMs of T2D patients on the other hand produced lower concentrations of IL-1RA. This finding is supported by others who show that whole blood from diabetes patients with latent TB, stimulated with TB antigens, produce lower concentrations of IL-1RA compared to whole blood from non-diabetes controls (51). CSF2 concentrations secreted by human MDMs was further shown to be negatively correlated with *M.tb* load in the macrophages (52). Despite the lower concentrations of CSF2 produced by MDMs of T2D patients, we did not observe changes in the bacterial loads of the cells.

We recognize the study limitations such as the small sample size which stems from the inclusion/exclusion criteria used in the study as well as the invasive nature of the bronchoscopy and voluntary participation in having the procedure done. All our participants were recruited in a single geographical setting. Changes observed in gene expression and DNA methylation may be different in other populations. QFT status may be a confounding factor in our analysis. Although it did not change the interpretation of the CFU data, we cannot rule out the potential effect QFT status may have on the gene expression and cytokine data. Our sample size was however too small to stratify for QFT status. Differences in phenotype between the two cell populations may be due to the two different platforms used (FACS vs CyTOF). Many factors affect HAM function including smoking and exposure to environmental smoke (half of the participants in each group were smokers), exposure to alveolar lining fluid and their interaction with epithelial and other immune cells. Given the small sample size, we could not assess the effect of smoking on HAM function. Although *ex vivo* HAM cultures do not fully recapitulate all factors affecting HAM function, it allows us to better understand intrinsic changes that occur in HAMs from T2D patients.

Take together we show here that early HAM functions were affected by T2D status resulting in a delay in gene expression, dysregulation in cytokine production driven by epigenetic changes and delays in neutrophil recruitment into the lung, which allow *M.tb* to better adapt to the host environment. We provide the first in-depth analysis of the diabetic HAM phenotype and function in the context of recent TB exposure providing a mechanistic explanation for increased TB susceptibility in T2D patients (Figure 8).

## Figures and Tables

**Table 1.** Characteristics of study participants.

Group	No-T2D (N=11)	T2D (N=12)	p-value

Age, mean ± SD	53.4 ± 6.3	54.2 ± 5.3	0.73
Sex, n (%)			
Female	10 (91)	11 (92)	1.00
Male	1 (9)	1 (8)	1.00
QFT status, n (%)			
Positive	11 (100)	7 (58.33)	**0.04**
Negative	0 (0)	5 (41.67)	**0.04**
BMI, mean ± SD	21.2 ± 4.3	27.1 ± 5.5	**0.01**
Clinical information, mean ± SD			
HbA1c (%)	5.2 ± 0.2	9.6 ± 1.8	**<0.01**
Total CHL (mmol/L)	4.8 ± 1.2	5.1 ± 1.1	0.56
Triglycerides (mmol/L)	1.1 ± 0.5	1.9 ± 1.1	**0.04**
HDL (mmol/L)	1.7 ± 0.3	1.4 ± 0.5	0.08
LDL (mmol/L)	2.6 ± 0.95	2.9 ± 0.97	0.69
Insulin (mIU/L)	11.8 ± 14.4	20.9 ± 40.9	0.50
Cortisol (nmol/L)	263.7 ± 64.5	266.1 ± 138.3	0.74
Known Diabetes, n (%)			
Yes	0 (0)	10 (83.33)	
No	11 (100)	2 (16.67)	
Smoking, n (%)			
Never smoked	4 (36.36)	6 (50)	0.68
Current smoker	7 (63.64)	6 (50)	0.68

Significant differences were determined using the Mann-Whitney U test for continuous variables and the Fisher Exact test for categorical variables. P<0.05 was considered statistically significant. QFT – QuantiFERON, BMI – body mass index, CHL – cholesterol, HDL – high-density lipoprotein, LDL – low-density lipoprotein.

**Table 2.** Relationship between host characteristics and early *M.tb* control (Day 1 CFU fold growth) in monocyte-derived macrophages (MDMs) and human alveolar macrophages (HAMs).

	
	*M.tb* growth in MDMs		*M.tb* growth in HAMs	
	
	Spearman r	p-value	Spearman r	p-value

BMI (kg/m2)	−0,1908	0,3833	0,2195	0,3523
HbA1c (%)	−0,0466	0,8329	0,4380	**0,0470**
Total CHL (mmol/L)	−0,3113	0,1481	0,8329	0,5912
Triglycerides (mmol/L)	−0,3027	0,1604	0,0143	0,9523
HDL (mmol/L)	0,1098	0,6181	0,0602	0,8010
LDL (mmol/L)	−0,2234	0,3056	−0,2812	0,2297
Insulin (mIU/L)	0,3098	0,1968	−0,1471	0,5862
Cortisol (nmol/L)	0,0689	0,7668	0,0105	0,9659

P<0.05 was considered statistically significant. BMI – body mass index, CHL – cholesterol, HDL – high-density lipoprotein, LDL – low-density lipoprotein.
